# Epidemiology and spatial distribution of *Echinococcus granulosus* in sheep and goats slaughtered in a hyperendemic European Mediterranean area

**DOI:** 10.1186/s13071-021-04934-9

**Published:** 2021-08-21

**Authors:** Antonio Bosco, Leucio Camara Alves, Paola Cociancic, Alessandra Amadesi, Paola Pepe, Maria Elena Morgoglione, Maria Paola Maurelli, Edyniesky Ferrer-Miranda, Kleber Régis Santoro, Rafael Antonio Nascimento Ramos, Laura Rinaldi, Giuseppe Cringoli

**Affiliations:** 1 Department of Veterinary Medicine and Animal Production, University of Naples Federico II, CREMOPAR, Naples, Campania Italy; 2 Department of Veterinary Medicine, Federal Rural University of Pernambuco, Recife, Pernambuco Brazil; 3 Centro de Estudios Parasitológicos y de Vectores (CEPAVE-CONICET-UNLP-asociado a CICPBA), La Plata, Buenos Aires, Argentina; 4 Laboratory of Molecular Biology, Federal University of Agreste of Pernambuco, Garanhuns, Pernambuco Brazil; 5 Laboratory of Parasitology, Federal University of Agreste of Pernambuco, Garanhuns, Pernambuco Brazil

**Keywords:** *Echinococcus granulosus*, Cystic echinococcosis, Sheep, Goats, Spatial distribution, Cysts

## Abstract

**Background:**

Cystic echinococcosis (CE) is a worldwide parasitic zoonosis caused by the larval stage of *Echinococcus granulosus* sensu lato affecting livestock, particularly sheep and goats. However, often this parasitosis is underestimated. For this reason, this study aimed to evaluate the epidemiological features and spatial distribution of CE in sheep and goats slaughtered in a hyperendemic Mediterranean area.

**Methods:**

A survey was conducted in the Basilicata region (southern Italy) from 2014 to 2019. A total of 1454 animals (1265 sheep and 189 goats) from 824 farms were examined for hydatid cyst detection by visual inspection, palpation and incision of target organs. All the CE cysts were counted and classified into five morphostructural types (unilocular, multiseptate, calcified, caseous and hyperlaminated). Molecular analysis was performed on 353 cysts. For spatial analysis, a kriging interpolation method was used to create risk maps, while clustering was assessed by Moran’s *I* test.

**Results:**

CE prevalence of 72.2% (595/824) and 58.4% (849/1454) was observed at the farm and animal levels, respectively, with higher values in sheep (62.9%) than goats (28.0%). The liver and lungs were the most frequently infected organs in both sheep and goats. Most of recovered cysts were of the calcified and multiseptate morphotypes. All the isolates were identified as *E. granulosus* sensu stricto (genotypes G1–G3). Spatial distribution showed a moderate clustering of positive animals.

**Conclusion:**

The findings of this study can be used to better understand the eco-epidemiology of echinococcosis and to improve CE surveillance and prevention programs in regions highly endemic for CE.

**Graphical abstract:**

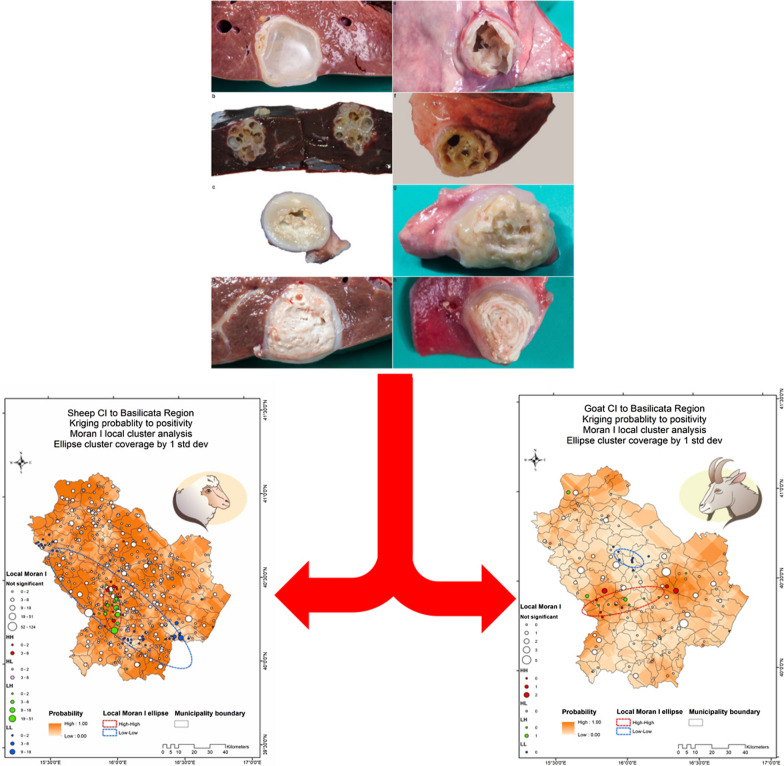

## Background

Cystic echinococcosis (CE) is a parasitic zoonosis caused by taeniid tapeworms, belonging to the *Echinococcus granulosus* sensu lato complex [1]. The domestic life cycle of this infection involves dogs as definitive hosts and a broad spectrum of mammals (e.g. sheep, goats, water buffalo, cattle) as intermediate hosts. Briefly, intermediate hosts become infected through ingestion of pasture grass contaminated with *E. granulosus* s.l. eggs released by infected dogs. The cycle is complete when definitive hosts ingest cysts (metacestodes) present in different organs (e.g. liver, lungs, spleen, heart) of infected intermediate hosts, particularly sheep and goats. Humans are considered accidental intermediate hosts [2].

Currently, *E. granulosus* s.l. complex comprises *E. granulosus* sensu stricto (genotypes G1–G3), *E. equinus* (G4), *E. ortleppi* (G5), *E. canadensis* (G6/G7, G8 and G10) and *E. felidis* [1, 3]. G1 is the most widespread genotype in intermediate hosts, as well as in human CE cases (88.4%) [4]. CE constitutes a significant financial constraint in the public health field and the livestock industry. The global burden of CE has been estimated at approximately 1 million disability-adjusted life-years (DALYs),and the world’s livestock industry loss has been estimated at around $3 billion a year [5, 6].

*Echinococcus granulosus* is a cosmopolitan species, but it is widespread in rural areas of central Asia, South America, and southern and eastern Europe [5, 7, 8]. The distribution of CE in different parts of the world is related to environmental and anthropogenic factors. Deplazes et al. [5] showed a heterogeneous geographic distribution in the European Mediterranean area, with prevalence values < 0.1% in the coastal regions of France and Spain, reaching > 50.0% in Italy, with a higher prevalence in the southern (Basilicata and Campania regions) and insular (Sardinia and Sicily) part of the country [8–10]. However, the reported prevalence of CE in livestock is widely underestimated, because the surveillance system based on reports recorded at slaughterhouses is still inefficient [9, 11]. In addition, surveillance system data are usually obtained for wide geographic areas without considering that the prevalence of CE can differ widely in different points of the same area [12]. Therefore, this study aimed to evaluate the epidemiological features and spatial distribution of CE in sheep and goat farms in a hyperendemic region of the European Mediterranean.

## Methods

### Study area and sampling

This study was carried out from 2014 to 2019 in the Basilicata region of southern Italy. This region comprises an area of about 10,000 km^2^, where the provinces of Potenza (40° 38′ N; 15° 48′ E) and Matera (40° 39′ N; 16° 36′ E) are located. The area is characterized by a Mediterranean climate with dry summers and rainfall concentrated between October and March. Precipitation is abundant, about 1200 mm/year [13]. The average temperature in the coldest month (January) is about +8 °C, and the warmest month (August) about +28 °C, with an annual average of +14 °C.

A geographic information system (GIS) of the Basilicata region was constructed using the administrative boundaries at the provincial and municipal levels as data layers. In order to uniformly sample the animals throughout the study area, the region was divided into 100 quadrants, by overlaying a grid of 10 × 10 km. In each quadrant, about 15 small ruminants aged 3–7 years from 8–9 farms were randomly selected, considering the farmer’s availability to collaborate. A total of 1454 animals (1265 sheep and 189 goats) from 824 farms from all 100 quadrants were examined. The geographical coordinates of each sheep and goat farm were obtained according to the farm code of each farm.

### Postmortem examination

The animals from the farms selected from all 100 quadrants during the study were transported to an abattoir for slaughter and postmortem inspection. For each animal slaughtered, CE detection was performed by visual inspection, palpation and incision of the heart, kidneys, liver, lungs and spleen. For each positive sheep, the CE cysts were counted and classified into five morphostructural types (unilocular, multiseptate, calcified, caseous and hyperlaminated) in accordance with Conchedda et al. [8].

When cystic lesions were attributable to CE, the animal and the farm to which it belonged were classified as positive.

### Molecular analysis

The molecular study was carried out on 353 cysts (300 from sheep and 53 from goats). At random, hydatid fluid (where present) or the parasitic membrane was obtained for molecular analysis [14]. From 300 sheep (179 from liver, 102 from lungs, 11 from spleen, eight from kidneys) and from all 53 goats, one cyst for each animal was collected (independently of the morphotype of cysts).

The cysts and the cystic liquid were collected and stored at –20 °C until DNA extraction. Genomic DNA was extracted using the QIAamp DNA Mini Kit (Qiagen, Hilden, Germany) [14]. Polymerase chain reaction (PCR) for the cytochrome *C* oxidase subunit 1 (*CO1)* gene was performed as reported in Capuano et al. [14], while PCR for the 12S rDNA gene was conducted as described in Rinaldi et al. [15]. PCR products were detected on a 2% ethidium bromide-stained low melting agarose gel (BIO-RAD, Spain) for both PCR reactions. Bands were cut from the gel under UV exposure, and the amplified DNAs were purified using the QIAquick Gel Extraction Kit(Qiagen, Germany). The PCR products were sequenced and analyzed using Chromas version 2.6.6 software. DNA sequences comparison was achieved using GenBank with the BLAST system and ClustalW.

### Geostatistical analysis

All georeferencing and data were expressed in geographical ETRS89 format and were projected to UTM zone 33N at reference datum WGS84, as specified by the RSDI [Regional Spatial Data Infrastructure] Basilicata Geoportale [16].

### Indicator kriging to access continuous area probability

Disease incidence detection and probability mapping were performed in three steps. The first step produced empirical semi-variograms, which represented half of the mean square difference between pairs of sampling locations (Eq. 1).

1 $$\gamma \left( h \right) = \frac{1}{2N\left( h \right)}\mathop \sum \limits_{i = 1}^{N\left( h \right)} \left[ {z\left( {x_{i} + h} \right) - z\left( {x_{i} } \right)} \right]^{2} { },$$

where *N*(*h*) is the number of data pairs for the lag *h*, while *h* is the distance between animal sampling sites and *z*(*xi*) is the location of the animal sample. The stable semi-variogram function [17] was used to fit the semi-variogram model to the empirical data.

The second step involved estimation mapping to predict the presence or absence of disease in an unknown location. Indicator kriging was used to estimate mapping distributions under a given threshold [18]. The resulting data were interpreted as values between zero and one. The greater the value, the more probable the occurrence of the event, i.e., higher probabilities indicate a greater likelihood of finding a farm with an infected animal.

The last step consisted of estimation mapping for the probability of presence or absence in the range 0–1, as described in Adhikary et al. [19].

### Local Moran’s *I* statistics for spatial autocorrelations and clustering

Local spatial autocorrelations were used to calculate the significance levels of local indicators of spatial association (LISA) based on observed farms throughout the study area.

In this study, LISA [20, 21] was used to reflect the degree of correlation between the incidence of disease among animals on a given farm and the incidence among animals on nearby farms. The local Moran’s *I* index was defined as:

$$I_{j} = \frac{{n\left( {X_{i} - \overline{X}} \right)\mathop \sum \nolimits_{i = 1}^{n} W_{ij} \left( {X_{j} - \overline{X}} \right)}}{{\mathop \sum \nolimits_{i = 1}^{n} \left( {X_{i} - \overline{X}} \right)^{2} }},$$

where *n* is the number of space units involved in the analysis, *X*_*i*_ and *X*_*j*_ represent the observed values of a phenomenon (or an attribute characteristic) *x* on the *I* and *j* of the space unit, and *W*_*ij*_ is the spatial weight generally based on a point distance function.

The *I*_*j*_ value can be mapped to highlight data based on relative importance and surrounding behavioral association, leading to four categories:

-Low-low (LL): a point with a low value with surrounding points with low values (positive *I*_*j*_* =* same behavior), interpreted as a “*cold spot cluster*”;

-High-high (HH): a point with a high value with surrounding points with high values (positive *I*_*j*_* =* same behavior), interpreted as a “*hot spot cluster*”;

-Low-high (LH): a point with a low value with surrounding points with high values (negative *I*_*j*_* =* different behavior), interpreted as a “*cold outlier spot*”;

-High-low (HL): a point with a high value with surrounding points with lower values (negative *I*_*j*_* =* different behavior), interpreted as a “*hot outlier spot*”.

These categories can facilitate a direct interpretation of behavioral phenomena over the entire area.

Based on the spatial location of the LL and HH points, two ellipses were constructed in order to approach its spatial dimensionality, using a distance between farms approach.

All these results were utilized to construct a map for the purpose of interpreting disease behavior in a whole view, providing a “complete picture.”

All analyses were performed using the ESRI ArcGIS ArcMap 10.6 software.

## Results

Overall, CE prevalence of 72.2% (595/824; 95% confidence interval [95% CI] 69.0–75.2%) and 58.4% (849/1454; 95% CI 55.8–60.9%) was found at the farm and animal levels, respectively. CE was higher in sheep (796/1265, 62.9%; 95% CI 60.3–65.5%) than goats (53/189, 28.0%; 95% CI 22.1–34.8%) (*P* < 0.0001).

**Fig. 1:**
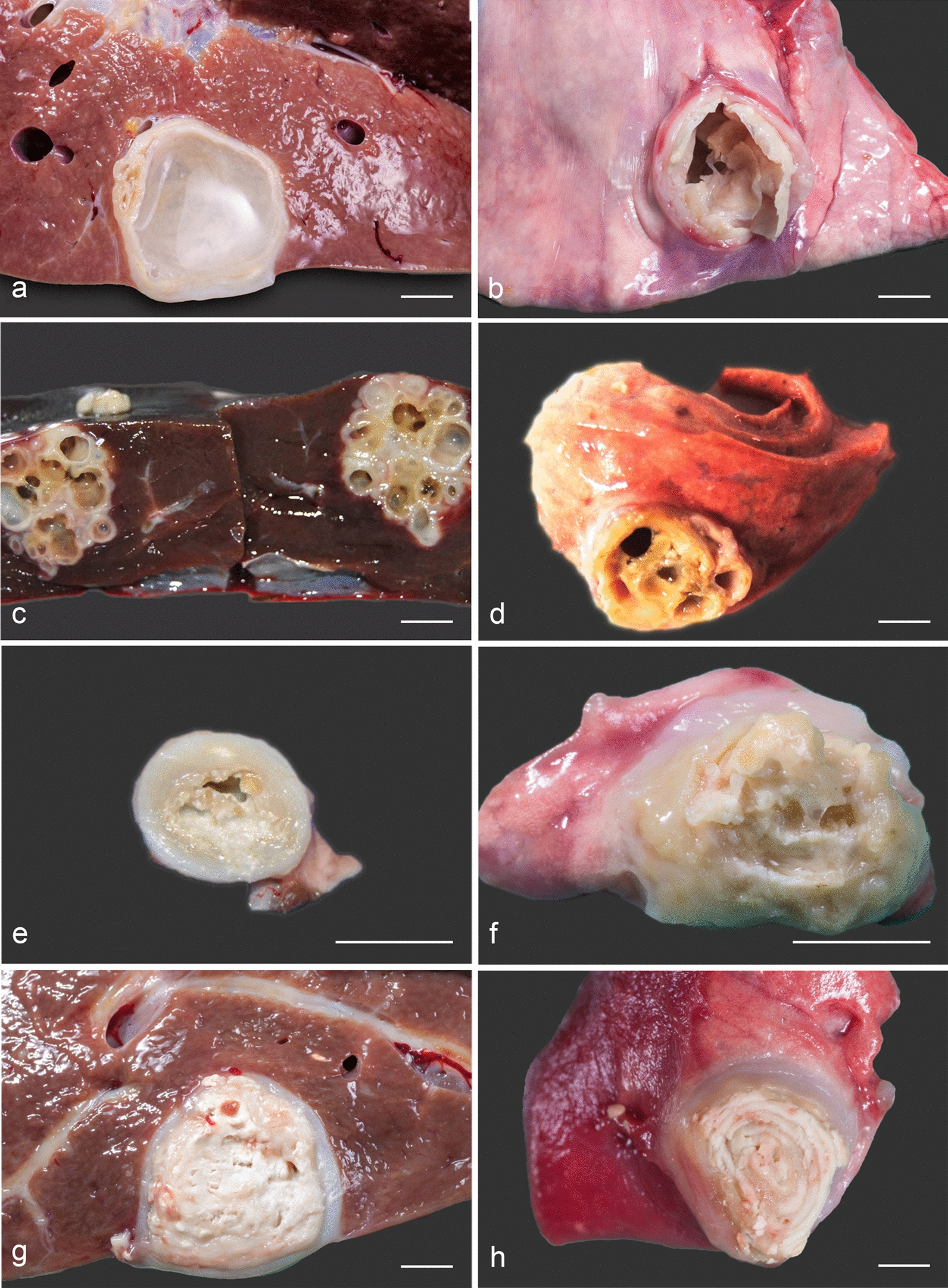
Cystic echinococcosis (CE) cyst morphotypes recovered from slaughtered sheep and goats. Unilocular cysts in liver (**a**) and lung (**b**) of sheep; multiseptate cysts with cavity divided by septa into spheroidal chambers of widely variable number in liver (**c**) and lung (**d**) of goat; calcified cyst showing almost virtual internal chambers in liver (**e**) and lung (**f**) of sheep; caseous cyst with cavity filled with a thick matrix of cheesy consistency in liver (**g**) of sheep; hyperlaminated cyst with the virtual cavity filled with sheets of laminated tissue in lung (**h**) of goat

Animals were found with one (39.7%), two (59.4%) or three (0.9%) infected organs. Regarding the organ distribution of CE, the liver and lungs were the most frequently infected visceral organs in sheep, as reported in Table 1. Very few sheep or goats (< 1%) had cysts in other organs (heart, spleen and kidneys) (Table 1). A total of 4577 cysts recovered from infected sheep and 229 cysts from infected goats were examined (Fig. 1). In the liver and lungs, the majority of the cysts belonged to the calcified and multiseptate morphotypes (Table 2). The molecular study allowed us to identify the presence of *E. granulosus* s.s. (GenBank U50464, M84662 and M84663 for *CO1* and GenBank AY462129 and DQ822451 for 12S) from ovine and caprine isolates. The G1 genotype was the most common in both sheep (70.0%; 95% CI 64.4–75.1%) and goats (79.3%; 95% CI 65.5–88.7%). No significant differences were found between genotypes and cyst morphotypes or localization of cysts (*P* > 0.05).

**Table 1:** Anatomical localization of cystic echinococcosis cysts in sheep and goats slaughtered

Organ	No. of positive animals; prevalence (%) (95% CI)	
	Sheep	Goats
Liver	671; 84.3 (81.6–86.7)	35; 66.0 (52.6–77.3)
Lungs	627; 78.8 (75.8–52.3)	25; 47.2 (34.4–60.3)
Spleen	11; 1.4 (0.7–2.4)	0; 0
Kidneys	8; 1.0 (0.5–1.9)	0; 0
Heart	4; 0.5 (0.2–1.2)	1; 1.9 (0.3–9.9)

Percentages were calculated in relation to the total number of infected sheep (*n* = 796) and goats (*n* = 53)

**Table 2:** Frequency of cystic echinococcosis cyst morphotypes recovered from each organ of sheep and goats slaughtered

Animal species	Organ	No. of cysts (%)					
		Unilocular	Multiseptate	Calcified	Caseous	Hyperlaminated	Total
Sheep	Liver	214 (7.9%)	592 (22.1%)	1099 (40.9%)	241 (8.9%)	536 (19.9%)	2682
	Lungs	168 (8.9%)	449 (23.9%)	729 (38.9%)	205 (10.9%)	321 (17.1%)	1872
	Spleen	0 (0%)	0 (0%)	0 (0%)	0 (0%)	11 (100%)	11
	Kidneys	0 (0%)	8 (80.0%)	0 (0%)	0 (0%)	0 (0%)	8
	Heart	0 (0%)	0 (0%)	3 (75.0%)	0 (0%)	1 (25.0%)	4
	Total						4577
Goats	Liver	12 (9.3%)	29 (22.5%)	48 (37.2%)	18 (13.9%)	22 (17.1%)	129
	Lungs	5 (5.1%)	19 (19.2%)	40 (40.4%)	14 (14.1%)	21 (21.2%)	99
	Spleen	0 (0%)	0 (0%)	0 (0%)	0 (0%)	0 (0%)	0
	Kidneys	0 (0%)	0 (0%)	0 (0%)	0 (0%)	0 (0%)	0
	Heart	0 (0%)	0 (0%)	1 (100%)	0 (0%)	0 (0%)	1
	Total						229

**Fig. 2:**
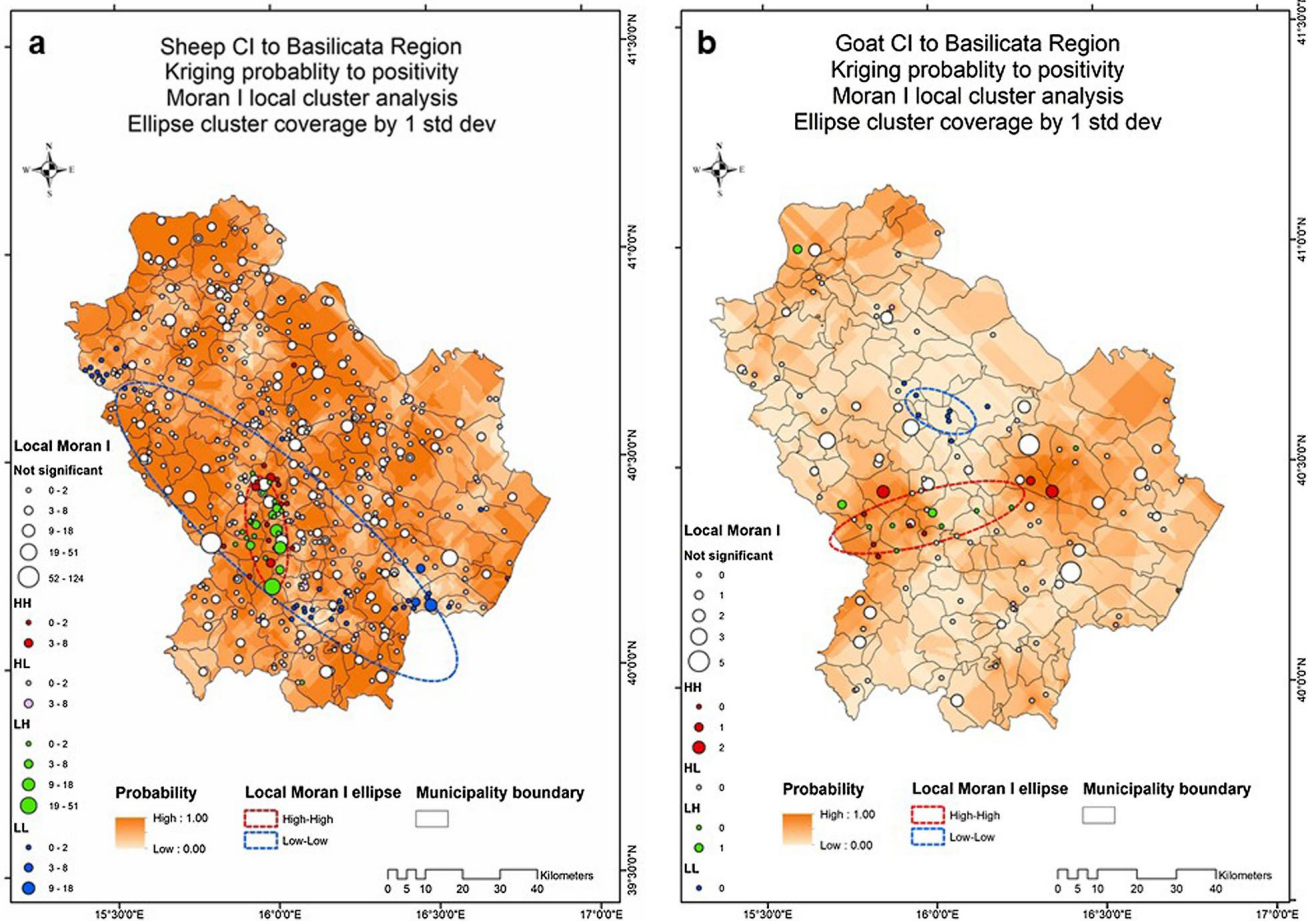
Kriging probability and local Moran’s *I* statistics for spatial autocorrelations and clustering in sheep (**a**) and goat farms (**b**). Darker colors indicate higher probability of finding a farm with an infected animal. Ellipses indicate the LL and HH clustering

A higher prevalence of positive animals was found in Potenza province. The spatial distribution of positive animals is shown in Fig. 2. Spatial distribution showed a moderate clustering of positive animals.

## Discussion

Data on the prevalence of CE in some Italian regions are scarce. In Basilicata, prevalence ranging between 5 and 28% was reported in sheep from 1996 to 2002 [22], and a value of 12% was estimated for the period 2010–2015 through a Bayesian analysis [9]. No previous data were available for goats. The prevalence of CE found in this study in the Basilicata region was 62.9% in sheep and 28.0% in goats. These values are higher than those reported in sheep and goats in other countries of the Mediterranean area, respectively: 30.2% and 7.6% in Greece [23]; 16.4% and 2.9% in Tunisia [24]; 6.9% and 1.6% in Algeria [25]; < 0.1% for both in Spain [26]; and < 0.002% for sheep and absence of infected goats in the last national census conducted in France [27]. The variation in the prevalence of CE in different parts of the world may be associated not only with environmental factors such as cool temperatures, high rainfall and shade, which increase the probability of egg survival in the environment and favor the transmission of CE in livestock, but also with control measures and breeding systems, number of dogs in each location, and education level and economic status of the population [28]. In the Mediterranean area, CE is predominant particularly in countries with large numbers of grazing sheep. Moreover, transmission is favored by farmers who feed shepherd dogs with infected viscera and by the lack of knowledge among the population about good prevention practices for this parasitosis [5, 29].

The results from the present study showed that the prevalence of CE was higher in Potenza than in Matera province. The total number of sheep and goat farms is higher in Potenza (4261 and 337, respectively) than in Matera (872 and 391, respectively) (source Regional DataBank: https://bdr.rete.basilicata.it/#/analytics). The kriging probability and Moran’s analysis also showed moderate clustering in the southwestern part of Potenza province. The climate in this zone is characterized by higher humidity, which could contribute to the persistence of the eggs in the environment. However, further precise studies will be required to gain a deeper understanding of the environmental differences between these provinces and factors that could favor the persistence of CE in this area.

The region also hosts a dog population of 92,208 animals (source Ministry of Health, canine registry), of which about 32.0% are shepherd dogs. The entire Basilicata region has a substantial sheep and goat farming tradition, usually based on extensive management using broad pastures. Therefore, dogs potentially infected with *E. granulosus* s.l. can contaminate the grazing pastures with feces containing eggs, contributing to the high prevalence of CE in livestock. For these reasons, the infection of small ruminants in this area is probably associated with various optimal conditions for the transmission of this parasite (e.g. high density of canine population, lack of dog deworming programs, inappropriate animal management practices by farmers).

Lastly, the higher prevalence of CE in sheep than in goats can be attributed to the areas where these animals graze, as sheep eat more grass from contaminated pastures [30]. Regarding the distribution of CE according to organ, the liver and lungs were the visceral organs most frequently infected among both sheep and goats, followed by the heart, spleen and kidneys. These findings agree with those of other authors, who found that the liver and lungs of sheep were commonly infected with CE [31–33]. However, some authors noted that the lung parenchyma has a spongy consistency and a greater capillary bed, which supports a higher presence of cysts, whereas the compact tissues of the liver resist the development of larger cysts [34, 35]. A precise characterization of cyst morphotypes is also very useful for the accurate evaluation of *E. granulosus* s.l. epidemiology in a specific territory [8]. The results of the present study revealed the presence of unilocular cysts (non-degenerate), which are potentially infectious for the definitive host and therefore enable the persistence of the parasite in the study area. Molecular results showed the presence of *E. granulosus* s.s. However, the study was limited in the small number of cysts analyzed (353/4806); nevertheless, the contribution of other species of *Echinococcus* in this area is very small, and therefore the results could be considered largely representative. Indeed, according to other studies performed in Italy [15, 36–39], *E. granulosus* s.s. is the most widespread species in ruminants in the country, and it must be rigorously controlled due to its recognized infectivity in humans [4].

Therefore, the areas with low and high clusters of cases identified in the present study (Fig. 2) can serve to identify hot spots for transmission of *E. granulosus* s.s. not only among sheep and goats but also for human infection. Indeed, the transmission of *E. granulosus* is favored by farmers who feed shepherd dogs with infected viscera, representing a source of risk for the human population [40]. In this way, the results from this spatiotemporal analysis on echinococcosis in sheep and goats revealed moderate clustered patterns for the period 2014–2019. However, further analyses are needed to better understand the eco-epidemiology of this parasite through the correlation between these clusters and the real risk factors of infection for animals and humans, in order to undertake effective control strategies.

This study is part of a research project concerning mapping of diseases caused by viral, bacterial and other parasitic infections found in ruminants in the Basilicata region using GIS. These maps are intended to be used in control programs to prevent and control CE in ruminants. In this context, a multidisciplinary program using a One Health perspective is required to control the transmission of *E. granulosus* and develop an educational program for farmers. Through the EchinoCamp project in the Campania region over the course of eight years [10], we demonstrated that a reduction in *E. granulosus* s.s. infection rates in dogs, humans and livestock (e.g. a decrease of up to 30% was observed in sheep) is feasible when synergistic monitoring activities for the control of CE are applied.

## Conclusions

The present study provides evidence of the persistence of CE in a hyperendemic European Mediterranean area. The identification of these disease hot-spot areas is important in order to understand the eco-epidemiology of echinococcosis and the persistence of infection, and thus to improve echinococcosis prevention programs and surveillance that will be important in reducing CE not only in animals, but also in humans. However, further studies are required to better understand the risk factors in hot-spot areas identified in this region, with the implementation of epidemiological studies in other intermediate hosts (e.g. cattle), as well as in definitive hosts.

## Data Availability

All data generated or analyzed during this study are included in this published article. The datasets analyzed during the current study are available from the corresponding author on reasonable request.

## Abbreviations

CE: Cystic echinococcosis
*Echinococcus granulosus * s.l.: *Echinococcus granulosus* sensu lato
*Echinococcus granulosus * s.s.: *Echinococcus granulosus* sensu stricto
GIS: Geographic information system
LL: Low–low
HH: High–high
LH: Low–high
HL: High–low
95% CI: 95% confidence interval

## References

[1] Maksimov P, Bergmann H, Wassermann M, Romig T, Gottstein B, Casulli A (2020). Species detection within the *Echinococcus granulosus sensu lato* complex by novel probe-based real-time PCRs. Pathogens.

[2] Budke CM, Casulli A, Kern P, Vuitton DA (2017). Cystic and alveolar echinococcosis: successes and continuing challenges. PLoS Negl Trop Dis.

[3] Vuitton DA, McManus DP, Rogan MT, Romig T, Gottstein B, Naidich A (2020). International consensus on terminology to be used in the field of echinococcoses. Parasite.

[4] Alvarez Rojas CA, Romig T, Lightowlers MW (2014). *Echinococcus granulosus sensu lato* genotypes infecting humans-review of current knowledge. Int J Parasitol.

[5] Deplazes P, Rinaldi L, Alvarez Rojas CA, Torgerson PR, Harandi MF, Romig T (2017). Global distribution of alveolar and cystic echinococcosis. Adv Parasitol.

[6] World Health Organization: Echinococcosis. 2021. https://www.who.int/news-room/fact-sheets/detail/echinococcosis/. Accessed 24 Mar 2021.

[7] Agudelo Higuita NI, Brunetti E, McCloskey C (2016). Cystic echinococcosis. J Clin Microbiol.

[8] Conchedda M, Seu V, Capra S, Caredda A, Pani SP, Lochi PG (2016). A study of morphological aspects of cystic echinococcosis in sheep in Sardinia. Acta Trop.

[9] Loi F, Berchialla P, Masu G, Masala G, Scaramozzino P, Carvelli A (2019). Prevalence estimation of Italian ovine cystic echinococcosis in slaughterhouses: a retrospective Bayesian data analysis, 2010–2015. PLoS ONE.

[10] Cringoli G, Pepe P, Bosco A, Maurelli MP, Baldi L, Ciaramella P (2021). An integrated approach to control cystic echinococcosis in southern Italy. Vet Parasitol.

[11] Cassini R, Simonato G, Mulatti P, Ravagnan S, Danesi P, Pascotto E (2019). A new approach to outbreak management for bovine cystic echinococcosis cases in hypo-endemic areas. Vet Parasitol Reg Stud Rep.

[12] Arezo M, Mujica G, Uchiumi L, Santillán G, Herrero E, Labanchi JL (2020). Identification of potential ‘hot spots’ of cystic echinococcosis transmission in the province of Río Negro. Argentina. Acta Trop.

[13] Caneva G, Fascetti S, Galotta G (1997). Aspetti bioclimatici e vegetazionali della costa tirrenica della Basilicata. Fitosociologia.

[14] Capuano F, Rinaldi L, Maurelli MP, Perugini AG, Veneziano V, Garippa G (2006). Cystic echinococcosis in water buffaloes: epidemiological survey and molecular evidence of ovine (G1) and buffalo (G3) strains. Vet Parasitol.

[15] Rinaldi L, Maurelli MP, Capuano F, Perugini AG, Veneziano V, Cringoli G (2008). Molecular update on cystic echinococcosis in cattle and water buffaloes of southern Italy. Zoonoses Public Health.

[16] RSDI Basilicata Geoportale. 2021. http://rsdi.regione.basilicata.it/. Accessed 24 Mar 2021.

[17] ESRI (2004). Using ArcGIS geostatistical analyst.

[18] Wacharapong S, Charoenjit K, Hrimpeng K, Jittimanee J (2020). Mapping the probability of detecting *Burkholderia pseudomallei* in rural rice paddy soil based on indicator kriging and spatial soil factor analysis. Trans R Soc Trop Med Hyg.

[19] Adhikary PP, Dash CJ, Chandrasekharan H, Rajput TBS, Dubey SK (2012). Evaluation of groundwater quality for irrigation and drinking using GIS and geostatistics in a peri-urban area of Delhi, India. Arab J Geosci.

[20] Anselin L (1995). Local Indicators of Spatial Association–LISA. Geogr Anal.

[21] Fotheringham AS, Brunsdon C, Charlton M (2002). Geographically weighted regression the analysis of spatially varying relationships.

[22] Garippa G, Battelli G, Cringoli G, Giangaspero A, Giannetto G, Manfredi MT (2004). Updating on animal echinococcosis in Italy. Parassitologia.

[23] Chaligiannis I, Maillard S, Boubaker G, Spiliotis M, Saratsis A, Gottstein B (2015). *Echinococcus granulosus* infection dynamics in livestock of Greece. Acta Trop.

[24] Lahmar S, Trifi M, Naceur SB, Bouchhima T, Lahouar N, Lamouchi I (2013). Cystic echinococcosis in slaughtered domestic ruminants from Tunisia. J Helminthol.

[25] Kouidri M, Benchaib-Khoudja F, Boulkaboul A, Selles SMA (2013). Cystic echinococcosis in small ruminants in Tiaret (Algeria). Glob Vet.

[26] Carmena D, Sánchez-Serrano LP, Barbero-Martínez I (2008). *Echinococcus granulosus* infection in Spain. Zoonoses Public Health.

[27] Umhang G, Richomme C, Bastid V, Boucher J-M, Peytavin de Garam C, Itié-Hafez S (2020). National survey and molecular diagnosis of *Echinococcus granulosus sensu lato* in livestock in France, 2012. Parasitology.

[28] Sánchez Thevenet P, Alvarez HM, Torrecillas C, Jensen O, Basualdo JA (2020). Dispersion of *Echinococcus granulosus* eggs from infected dogs under natural conditions in Patagonia, Argentina. . J Helminthol.

[29] Otero-Abad B, Torgerson PR (2013). A systematic review of the epidemiology of echinococcosis in domestic and wild animals. PLoS Negl Trop Dis.

[30] Ibrahim MM (2010). Study of cystic echinococcosis in slaughtered animals in Al Baha region, Saudi Arabia: interaction between some biotic and abiotic factors. Acta Trop.

[31] Nyero D, Zirintunda G, Omadang L, Ekou J (2015). Prevalence of hydatid cysts in goats and sheep slaughtered in Soroti Municipal Abattoir Eastern Uganda. Afr J Parasitol Res.

[32] Conchedda M, Seu V, Capra S, Caredda A, Pani SP, Lochi PG (2012). Cystic echinococcosis in sheep in Sardinia. Changing pattern and present status. Acta Trop.

[33] Assefa H, Mulate B, Nazir S, Alemayehu A (2015). Cystic echinococcosis amongst small ruminants and humans in central Ethiopia. Onderstepoort J Vet Res.

[34] Torgerson PR (2003). The use of mathematical models to simulate control options for echinococcosis. Acta Trop.

[35] Beigh AB, Darzi MM, Bashir S, Kashani B, Shah A, Shah SA (2017). Gross and histopathological alterations associated with cystic echinococcosis in small ruminants. J Parasit Dis.

[36] Busi M, Snábel V, Varcasia A, Garippa G, Perrone V, De Liberato C (2007). Genetic variation within and between G1 and G3 genotypes of *Echinococcus granulosus* in Italy revealed by multilocus DNA sequencing. Vet Parasitol.

[37] Casulli A, Manfredi MT, La Rosa G, Cerbo AR, Genchi C, Pozio E (2008). *Echinococcus ortleppi* and *E. granulosus* G1, G2 and G3 genotypes in Italian bovines. Vet Parasitol.

[38] Poglayen G, Varcasia A, Pipia AP, Tamponi C, Parigi M, Marchesi B (2017). Retrospective study on cystic echinococcosis in cattle of Italy. J Infect Dev Ctries.

[39] Varcasia A, Dessì G, Lattanzio S, Marongiu D, Cuccuru C, Carta S (2020). Cystic echinococcosis in the endemic island of Sardinia (Italy): has something changed?. Parasitol Res.

[40] Kachani M, Heath D (2014). Dog population management for the control of human echinococcosis. Acta Trop.

